# Bone Marrow-Derived Cell Accumulation in the Spinal Cord Is Independent of Peripheral Mobilization in a Mouse Model of Amyotrophic Lateral Sclerosis

**DOI:** 10.3389/fneur.2017.00075

**Published:** 2017-03-08

**Authors:** Kyle Peake, John Manning, Coral-Ann Lewis, Kevin Tran, Fabio Rossi, Charles Krieger

**Affiliations:** ^1^Department of Biomedical Physiology and Kinesiology, Simon Fraser University, Burnaby, BC, Canada; ^2^The Biomedical Research Centre, University of British Columbia, Vancouver, BC, Canada; ^3^Division of Neurology, Department of Medicine, Neuromuscular Disease Unit, VHHSC, Vancouver, BC, Canada

**Keywords:** busulfan, treosulfan, amyotrophic lateral sclerosis, bone marrow-derived cells, monocyte, granulocyte colony-stimulating factor, AMD3100, central nervous system

## Abstract

Bone marrow-derived cells (BMDCs) are capable of migrating across the blood–brain barrier (BBB) and accumulating in the central nervous system (CNS) when transplanted into recipients conditioned with whole-body irradiation or chemotherapy. We used the chemotherapeutic agents busulfan and treosulfan to condition recipient mice for transplantation with bone marrow (BM) cells isolated from donor mice ubiquitously expressing green fluorescent protein. We attempted to increase the accumulation of BMDCs in the CNS by mobilization of BMDCs using either, or both, granulocyte colony-stimulating factor (GCSF) or plerixafor (AMD3100). We also used several concentrations of busulfan. We hypothesized that higher concentrations of busulfan and BMDC mobilization would increase numbers of GFP^+^ cells in the CNS. The doses of busulfan employed (60–125 mg/kg) all resulted in high levels of sustained chimerism (>85% 1 year post-transplant) in both the blood and BM of wild-type (WT) mice and an amyotrophic lateral sclerosis (ALS) mouse model. Moreover, cells accumulated within the CNS in a dose-, time-, and disease-dependent manner. Conditioning with the hydrophilic busulfan analog treosulfan, which is unable to cross the BBB efficiently, also resulted in a high degree of BM chimerism. However, few GFP^+^ BMDCs were found within the CNS of WT or ALS mice of treosulfan-conditioned mice. Mobilization of BMDCs into the circulation using GCSF and/or AMD3100 did not lead to increased accumulation of GFP^+^ BMDCs within the CNS of WT or ALS mice. Weekly analysis of BMDC accumulation revealed that BMDCs accumulated more rapidly and to a greater extent in the CNS of ALS mice conditioned with a high dose (125 mg/kg) of busulfan compared to a lower dose (80 mg/kg). The number of GFP^+^ BMDCs in the CNS labeling with the proliferation marker Ki67 increased in parallel with BMDC accumulation within the CNS. Our results indicate that establishment of high levels of blood and BM chimerism alone is not sufficient to induce BMDC accumulation within the CNS and that CNS conditioning is a crucial requirement for BMDC accumulation to occur. Moreover, it appears that proliferation of BMDCs that infiltrate the CNS is partly responsible for cell accumulation in busulfan-conditioned ALS mice.

## Introduction

Following bone marrow (BM) transplantation (BMT), donor BM-derived cells (BMDCs) can migrate to various sites in the recipient including the BM and the central nervous system (CNS) (1). It is generally believed that the BMDCs that accumulate within the CNS are predominantly of monocytic lineage (2–4). These BM-derived monocytic lineage cells integrate within the CNS, exhibit several microglial markers, and have morphologies reminiscent of endogenous microglia and perivascular macrophages associated with blood vessels (2). Recent studies have shown that some populations of macrophages at CNS interfaces, such as perivascular macrophages, have stable populations and do not necessarily derive from BMDCs (5).

For successful BMT, niche space must be generated within the recipient BM compartment to allow for donor cell engraftment. While this is most commonly achieved using myeloablative irradiation, this procedure can lead to lethal damage and inflammation in the CNS as well as immunosuppression that increases the potential for secondary infections (6). Consequently, a number of recent reports have explored the utility of non-irradiative conditioning protocols. Busulfan (BU) is a clinically approved bifunctional alkylating agent that depletes non-cycling primitive stem cells in the BM and has weak immunosuppressive properties (7). We and others have found that conditioning with BU prior to BMT leads to high levels of BM chimerism and accumulation of BMDCs within the CNS (2–4, 8–12). In fact, Wilkinson et al. have claimed that BU conditioning enhances engraftment of BMDCs compared to whole-body lethal irradiation when using a higher dose of BU (125 mg/kg), than we and others have used previously (4).

The microglial population is established by primitive myeloid cells during embryogenesis and is largely self-sustaining in the adult CNS (13). In parabiotic models where chimerism is established physiologically by surgically connecting the circulations of two mice, rather than by injecting whole BM into the circulation, accumulation of donor BMDCs in the CNS does not occur even after irradiation of the parabiotic recipient (14). Similarly, transplantation of BMDCs following whole-body irradiation does not lead to accumulation of BMDCs in the CNS if the brain is protected by shielding, demonstrating the importance of brain conditioning for BMDC accumulation to occur in the CNS (15). Together, these observations suggest that reconstitution of the CNS with BMDCs requires both the presence of monocytic lineage progenitors in the blood circulation capable of efficient transmigration across the blood–brain barrier (BBB), and conditioning of the BBB/CNS, possibly through ablation of endogenous proliferating microglia (8).

Given the potential utility of BMDCs as a vehicle to deliver therapeutics to the CNS, we sought to further elucidate the mechanisms involved in BMDC accumulation within the CNS of a mouse model of amyotrophic lateral sclerosis (ALS). ALS is a neurogenerative disease characterized by the progressive loss of motoneurons in the brainstem and spinal cord, as well as neuron loss in the cerebrum. ALS pathogenesis is heterogeneous and is associated with mutations in several genes, as well as with other pathological processes including excitotoxicity, oxidative injury, protein aggregation, and altered RNA metabolism that result in cellular dysfunction (16). The gene mutations found in ALS include those for Cu/Zn superoxide dismutase (SOD1), which subsequently led to the development of transgenic mice over-expressing human mutant SOD1 (mSOD) as a murine model of ALS (17). The G93A mSOD transgenic model develops progressive motoneuron degeneration and limb paralysis mimicking human ALS.

Regardless of the initiating causes of ALS, pathologically ALS is characterized by activation and proliferation of microglia, in addition to the neuron loss, suggesting that microglia contribute to ALS pathogenesis (16). Recent work has claimed that the increased number of monocyte lineage cells found in the spinal cords of mSOD mice, and possibly ALS patients, is due to recruitment of circulating Ly6C^hi^ monocytes (18). Butovsky and colleagues reported that recruited Ly6C^hi^ monocytes were detected in the spinal cord early in disease and that the number of Ly6C^hi^ cells increased as disease progressed, reflecting further recruitment of monocytes. By contrast, the population of resident microglia, identified using the marker CD39, decreased with disease progression (18). Use of anti-Ly6C monoclonal antibody against infiltrating monocytes slowed disease progression, presumably by decreasing the entry of recruited Ly6C monocytes to the spinal cord (18). However, using RNA sequencing and evaluation of specific expression patterns of spinal cord microglia and peripheral monocytes, Chiu et al. found resident microglia increased in number during disease progression, but that the monocyte population did not increase, unlike the results of Butovsky et al. (19). Furthermore, Chiu et al. did not observe many Ly6C^hi^ cells in spinal cord, suggesting limited infiltration of peripheral monocytes (19). In previous studies where we generated parabiotic mice pairs using wild-type (WT) mice ubiquitously expressing green fluorescent protein (GFP) and mSOD mice, we did not observe any entry of GFP^+^ cells from the parabiont into the CNS of the mSOD mouse, suggesting that there is no infiltration of monocytes into the CNS of mSOD mice under physiological conditions (14). Notably, parabiosis cannot be maintained when the mSOD mouse becomes very debilitated, limiting the evaluation of peripheral monocyte recruitment in late diseases stages.

In the present study, we transplanted donor BM cells isolated from mice ubiquitously expressing GFP in order to monitor and characterize BMDC accumulation in BU-conditioned mice. Using this transplantation model, we evaluated the effects that different doses of BU had on BMDC accumulation within the CNS. The current literature suggests that both circulating progenitors and CNS conditioning are required for BMDC accumulation within the CNS. As such, we also monitored the effects of pharmacological mobilization of BMDCs, and the consequences of minimizing CNS conditioning by using the BU analog treosulfan (TREO), on BMDC accumulation within the CNS. We had three hypotheses; we expected that BMDC accumulation would be dependent on the conditioning method and that BMDC accumulation in the spinal cord would increase with time, consequent to the proliferation of the BMDC within the CNS. We also hypothesized that mobilization of BMDC from BM would increase the number of monocyte lineage cells entering the spinal cord and contributing to BMDC accumulation.

## Materials and Methods

### Ethics Statement

All protocols related to the use of animals in this study were reviewed and approved by the University Animal Care Committee of Simon Fraser University and were in compliance with the Canadian Council of Animal Care, the NIH Guide for the Care and Use of Laboratory Animals, and the EEC Council Directive.

### Animals

A colony of transgenic mice that over-express mutant human superoxide dismutase-1 (mSOD; B6.Cg-Tg(SOD1*G93A)1Gur/J; Jax 004435) was established at Simon Fraser University using breeding pairs obtained from Jackson Laboratories (Bar Harbor, ME, USA). The mice were maintained under temperature-controlled conditions with a 12-h light:12-h dark cycle, and were supplied with food and water *ad libitum*. As mSOD^+^ females are unable to successfully breed, WT females (C57BL/6J; Jax 000664) were paired with mSOD^+^ males. Genotypes of mice were determined by PCR analysis of genomic DNA isolated from ear tissue collected during notching as described previously (17). The mSOD mice develop progressive motoneuron degeneration, culminating in muscle atrophy and eventually hind limb paralysis. Unless otherwise stated, mSOD mice, as well as age- and sex-matched controls, were collected at advanced stage of disease progression defined as >5 s to right from lateral recumbency.

Donor mice ubiquitously expressing GFP under the control of the β-actin promoter (C57BL/6; GFP/CD45.2) were obtained from Dr. I. Weissmann. Mice were bred and maintained as heterozygotes at Simon Fraser University, with genotypes being confirmed by observing GFP expression in collected ear tissue by fluorescence microscopy.

### Myelosuppressive Conditioning

The myelosuppressive dialkylating agent busulfan (Busulfex, Otsuka Pharmaceuticals, Japan) was diluted from pharmaceutical stock solution to 3 mg/mL with sterile PBS just prior to administration. Fractionated doses of 20 mg/kg/day of BU were administered *via* intraperitoneal injections until a total dose of 60–100 mg/kg was delivered as we have done previously (3, 11, 12). For a total dose of 125 mg/kg, fractionated doses of 25 mg/kg/day were administered for 5 days (4). BMTs were conducted 24 h following the final injection of BU.

Treosulfan (TREO; Medac, DE), a hydrophilic analog of BU that does not readily cross the BBB (20, 21), was resuspended in sterile ddH_2_O at a concentration of 50 mg/mL just prior to administration. Doses were selected based upon previous studies by Van Pel et al. and Nasa et al. (22, 23). Mice conditioned with 4,500 mg/kg TREO received daily intraperitoneal injections of 1,500 mg/kg/day over the course of 3 days while mice conditioned with 6,000 mg/kg TREO received daily intraperitoneal injections of 2,000 mg/kg/day over the course of 3 days. BMTs were conducted 72 h following the final injection of TREO as evidence suggests this improves grafting efficiency (23).

### Bone Marrow Transplantation

Bone marrow cells were isolated from the femurs and tibiae of GFP^+^ donor mice. Briefly, donors were euthanized, and the femurs/tibiae were removed and cleaned of tissue. The end caps were shaved off the bones, and BM cells were isolated by flushing the medullary cavity with sterile PBS. Red blood cells were lysed with ACK lysing buffer (A10492-01, Life Technologies), and the BM cells were resuspended in sterile PBS at a final concentration of 5 × 10^6^ cells/mL. Three hundred microliters of cell suspension was injected *via* the tail vein into conditioned recipients. Details of the BMT procedure are described elsewhere (11).

### Measuring Extent of Chimerism

In order to estimate the extent of BM chimerism, blood was sampled from the saphenous vein and red blood cells were lysed with ACK lysing buffer. Myeloid cells were labeled with anti-CD11b-APC (1:400; 17-0112-81, eBioscience) and anti-GR1-APC (1:400; 553129, BD Pharmingen) antibodies, while lymphoid cells were labeled with anti-CD3e-PECy7 (1:200; 25-0031-81, eBioscience) and anti-CD45R-PECy7 (1:200; 25-0452-81, eBioscience) antibodies. Cells were quantified by flow cytometry using a Guava flow cytometer (EMD Millipore, DE, USA) and analyzed using FlowJo (FlowJo, Ashland, OR, USA).

During tissue collection, a single femur was removed following perfusion with PBS and prior to fixation with paraformaldehyde. BM cells were flushed from the femur using FACS buffer (2mM EDTA + 2% fetal bovine serum in PBS) and subsequently processed in a similar fashion as blood samples above.

### Mobilization Experiments

Bone marrow cells were mobilized into the circulation using either granulocyte colony-stimulating factor (GCSF), AMD3100 (plerixafor), or a combination of GCSF and AMD3100. For GCSF-mediated mobilization, GCSF [Neupogen (filgrastim), Amgen, Thousand Oaks, CA, USA] was diluted from the pharmaceutical stock to 30 μg/mL with sterile PBS + 0.1% BSA, and mice received daily intraperitoneal injections of 300 μg/kg GCSF for five consecutive days (24). For AMD3100-mediated mobilization, AMD3100 octahydrochloride hydrate (A5602, Sigma) was resuspended to 0.5 mg/mL with sterile PBS, and mice received a single intraperitoneal injection of 5 mg/kg AMD3100 (25, 26). To combine mobilization treatments, mice received daily intraperitoneal injections of 300 μg/kg GCSF for 4 days followed by a single intraperitoneal injection of 5 mg/kg AMD3100 on day 5 (25, 26).

### Tissue Collection and Processing

Tissue was collected as previously described (27). Briefly, mice were euthanized and transcardially perfused with 30 mL of PBS. The femur was removed to determine chimerism, and subsequently, the mice were perfused with 30 mL of 4% paraformaldehyde (w/v). Spinal columns were dissected and post-fixed in 4% paraformaldehyde at 4°C overnight. The spinal cord was removed from the spinal column, and the tissue was then cryoprotected in 20% sucrose (w/v) for 24–72 h at 4°C. Tissue was then embedded in TissueTek OCT embedding compound (Sakura Finetek, USA), wrapped in parafilm, and stored at −80°C. Tissue was processed for immunohistochemistry as described previously (27). Briefly, 30 μm sections of lumbar region of the spinal cord were sectioned on a cryostat and every fifth section was collected, with at least 10 sections per sample. Samples were stored in DeOlmos solution at −20°C until immunohistochemical staining was performed.

### Assessment of Cell Proliferation

The cell proliferation-associated protein Ki67 was detected with anti-Ki67 antibody (1:1,000; ab15580, Abcam) using the free-floating immunohistochemistry procedure (2). Sections were mounted on slides using Vectashield mounting medium (Vector Labs, Burlingame, CA, USA), and proliferating cells were quantified in three individual lumbar spinal cord sections separated by at least 150 μm using a Leica epifluorescence microscope. EdU (5-ethynyl-2′-deoxyuridine; A10044, Thermo Fisher Scientific) was resuspended at a concentration of 5 mg/mL and administered to mice *via* intraperitoneal injections of 50 mg/kg EdU 48 and 24 h prior to tissue collection. Incorporation of EdU into proliferating cells was quantified using the Click-iT EdU Imaging Kit (C10640, Thermo Fisher Scientific) according to the manufacturer’s instructions. Iba1 (1:1,000, 019-19741, Wako) was labeled using the free-floating immunohistochemistry procedure (2), and Iba1^+^ cells were quantified in one half of three individual lumbar spinal cord sections separated by at least 150 μm using a Leica epifluorescence microscope.

### BMDC Quantification and Morphological Analysis

Spinal cord sections were mounted on slides using Vectashield mounting medium and analyzed using a Leica epifluorescence microscope. GFP^+^ cells were quantified in five individual lumbar spinal cord sections separated by at least 150 μm as we have done previously (2, 3, 12, 27). The morphology of the GFP^+^ cells was classified according to Vallières and Sawchenko (28). Briefly, “round cells” were round in shape with a diameter <9 μm; “rod cells” were oblong in shape with rounded ends and a length of ~20 μm; “elongated cells” had a length >20 μm without rounded ends; “ameboid” cells had a variable shape with a diameter >9 μm; and “stellate cells” had a small cell body and the presence of multiple ramified processes.

### Statistical Methods

All of the statistical analysis related to cell proliferation were carried out using SAS statistical software (version 9.4; SAS Canada, Toronto, ON, Canada). Analysis of GFP^+^ cell numbers and count data was used to compare the mean responses between treatments over time. The effects of treatment, time, and their two-way interaction were considered to be fixed effects in the model. *Post hoc* tests using the Tukey–Kramer method were used to locate differences in mean responses between pairs of treatments and/or timepoints. All of the model diagnostics relating to the residuals were verified. In cases where the residuals failed to satisfy the model assumptions, a natural logarithm transformation was used. Statistical analyses of cell accumulation data were conducted using SPSS software.

## Results

### BMDCs Accumulate within the Lumbar Spinal Cord of BU-Conditioned Mice in a Time-, Dose-, and Disease-Dependent Manner

**Figure 1:**
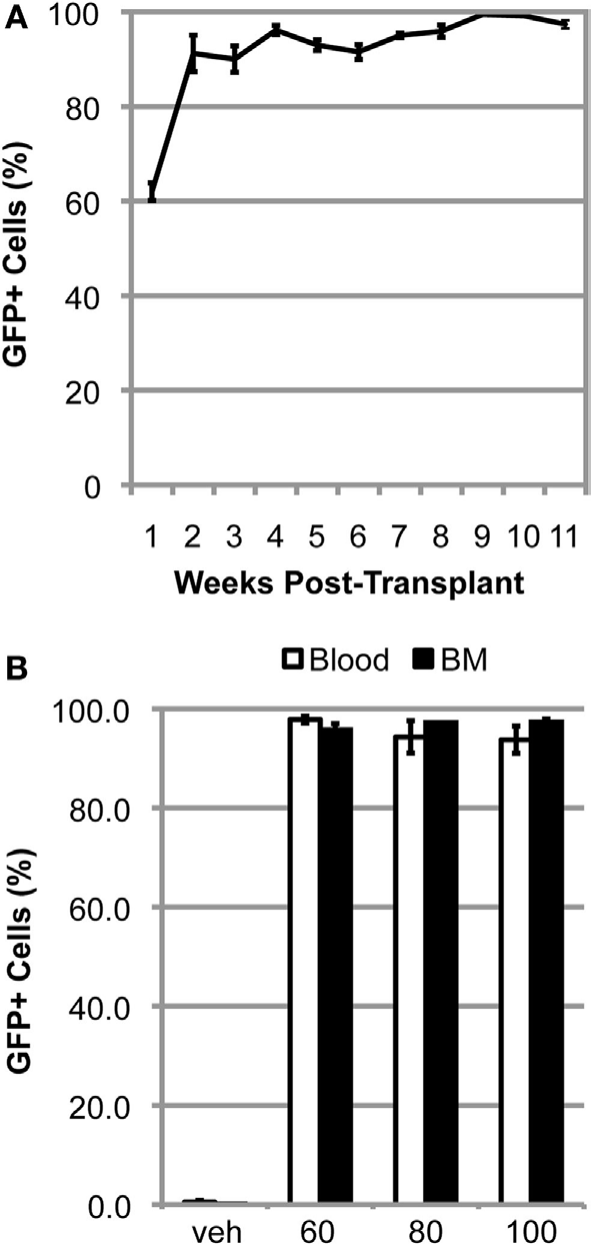
**High levels of chimerism are rapidly established and sustained in BU-conditioned mice**. **(A)** Weekly flow cytometric quantification of GFP^+^ cells within the blood in mutant SOD1 mice conditioned with 125 mg/kg of BU and transplanted with GFP^+^ donor bone marrow (BM) cells. **(B)** Flow cytometric quantification of GFP^+^ cells within the blood and BM 1-year post-bone marrow transplantation in wild-type mice conditioned with the indicated dose of BU. Data = mean ± SEM for *n* = 3 mice.

We have previously shown that myelosuppressive conditioning with 60–100 mg/kg BU, followed by BMT, was sufficient to generate mice with >80% BM chimerism after 11 weeks post-transplant (3). When mice are conditioned with 125 mg/kg BU, chimerism was rapidly established during the first few weeks post-BMT and a high degree of chimerism is maintained over the subsequent weeks in the blood (Figure 1A). The high level of chimerism achieved was stable, being maintained in the blood and BM of BU-conditioned WT mice for at least 1 year post-BMT (Figure 1B). Notably, there were no differences in the levels of chimerism achieved in the blood and BM with any of the concentrations of BU used to condition the mice (Figure 1B).

**Figure 2:**
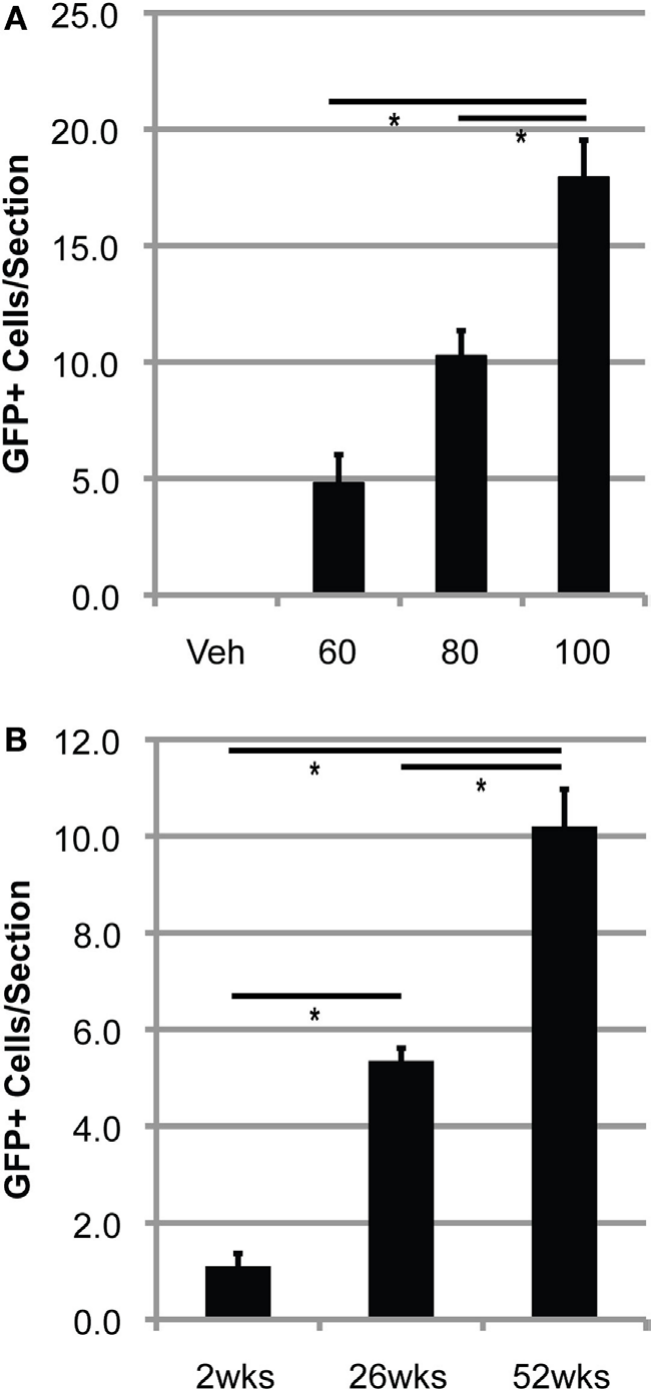
**Bone marrow-derived cells accumulate in the lumbar spinal cord of BU-conditioned mice in a time- and dose-dependent manner**. **(A)** Quantification of GFP^+^ donor cells within the lumbar spinal cord 1 year post-bone marrow transplantation (BMT) in wild-type (WT) mice conditioned with the indicated dose of BU (milligrams per kilogram). **(B)** Quantification of GFP^+^ donor cells within the lumbar spinal cord in WT mice conditioned with 80 mg/kg of BU and collected at the indicated times post-BMT. Data = mean ± SEM for *n* = 3 mice/group; data were analyzed by ANOVA with *p* < 0.05.

**Figure 3:**
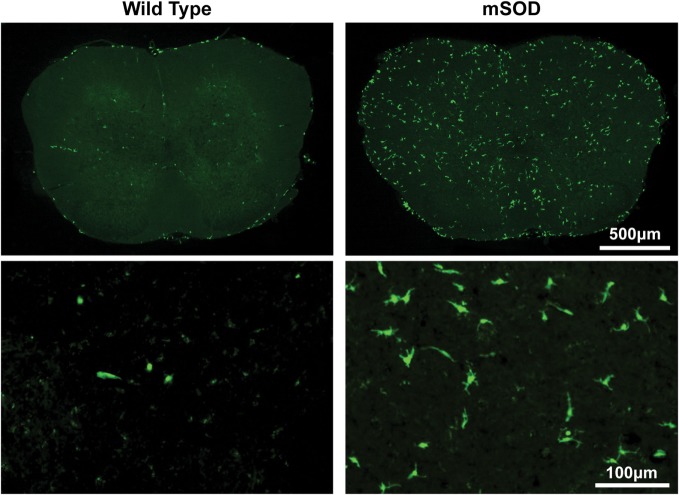
**Bone marrow-derived cells accumulate to a much greater extent in the lumbar spinal cord of BU-conditioned mutant SOD1 (mSOD) mice compared to wild-type (WT) controls**. Immunohistochemical analysis of lumbar spinal cord sections from late stage mSOD mice and age-matched WT controls that were conditioned with 125 mg/kg BU and transplanted with GFP^+^ donor bone marrow cells at 6–8 weeks of age. GFP^+^ BMDCs are shown in green. Results are representative images from *n* ≥ 3 mice.

Conditioning with 60–100 mg/kg BU allowed for accumulation of transplanted BMDCs within the lumbar spinal cord of WT mice (Figure 2). BMDC accumulation increased with BU dosage, whereas accumulation was absent in mice conditioned with vehicle (Figure 2A). Similarly, BMDCs accumulated within the lumbar spinal cord of BU-conditioned WT mice in a time-dependent manner for at least 1 year post-BMT (Figure 2B). Mice over-expressing mutant human Cu/Zn superoxide dismutase-1 (mSOD) are the most commonly used mouse model of ALS, recapitulating many features of the human disease including neuroinflammation and microgliosis (17). BU conditioning led to a much greater accumulation of BMDCs within the lumbar spinal cord of mSOD mice compared to controls, particularly when mice were conditioned with 125 mg/kg BU (Figure 3; Table 1). As BU-conditioned mSOD and WT mice had comparable levels of BM chimerism, this suggests that ALS disease-related signals enhance BMDC accumulation within the CNS.

**Table 1:** **Quantification of GFP^+^ donor cells within the lumbar region of the spinal cord in late stage mutant SOD1 (mSOD) mice and age-matched wild-type (WT) controls transplanted with GFP^+^ donor bone marrow cells at 6–8 weeks of age**.

	GFP^**+**^ cells/lumbar spinal section					
Treatment group	Total	Round	Rod	Elongated	Ameboid	Stellate
80 mg/kg BU WT (*n* = 3)	4.5 ± 1.0	1.4 ± 0.3 (31.1%)	1.2 ± 0.3 (26.7%)	1.3 ± 0.3 (28.1%)	0.4 ± 0.0 (8.9%)	0.2 ± 0.1 (4.4%)
80 mg/kg BU mSOD (*n* = 3)	55.1 ± 10.7	15.0 ± 4.0 (27.2%)	7.3 ± 0.7 (13.2%)	19.9 ± 3.4 (36.2%)	5.4 ± 1.9 (9.8%)	7.5 ± 1.1 (13.7%)
125 mg/kg BU WT (*n* = 5)	12.5 ± 1.5	1.1 ± 0.3 (8.6%)	5.2 ± 0.7 (41.3%)	5.9 ± 1.0 (47.0%)	0.0 ± 0.0 (0.0%)	0.4 ± 0.1 (2.9%)
125 mg/kg BU mSOD (*n* = 4)	117.9 ± 9.1	3.4 ± 0.4 (2.9%)	25.3 ± 2.3 (21.5%)	25.6 ± 2.5 (21.7%)	13.1 ± 0.4 (11.1%)	50.5 ± 3.8 (42.8%)
6,000 mg/kg TREO WT (*n* = 3)	4.7 ± 0.4	1.6 ± 0.3 (34.0%)	1.7 ± 0.3 (36.9%)	1.4 ± 0.5 (29.8%)	0.0 ± 0.0 (0.0%)	0.0 ± 0.0 (0.0%)
6,000 mg/kg TREO mSOD (*n* = 4)	17.0 ± 1.6	0.5 ± 0.2 (2.6%)	9.7 ± 0.7 (57.1%)	6.5 ± 0.9 (38.2%)	0.2 ± 0.1 (0.9%)	0.2 ± 0.1 (0.9%)

The morphology of BMDCs can provide insight into both the localization and activation state of the cells. Morphologies were determined according to the criteria outlined by Vallières and Sawchenko, whereby cells were classified as being round, rod, elongated, ameboid, or stellate (28). Generally, cells with stellate and ameboid morphologies are parenchymal in localization while round, rod, and elongated cells are usually located in perivascular regions (28). Very few parenchymal cells were detectable in BU-conditioned WT mice, even when 125 mg/kg was used, suggesting that few cells were able to actually infiltrate the CNS under these conditions (Table 1; Figure 3). Conversely, BU conditioning of mSOD mice led to significantly more BMDC accumulation within the lumbar spinal cord. This was particularly evident when 125 mg/kg BU was used, leading to >50% of the BMDCs accumulating within the lumbar spinal cord exhibiting morphologies that suggest parenchymal localization, with stellate cells being most prevalent (>40%; Table 1; Figure 3). Virtually all of the GFP^+^ BMDCs labeled with Iba1 and CD169, with the only exceptions being a small fraction of round/rod shaped cells (data not shown). These findings suggest that disease-related signals are required, along with BU conditioning, in order for BMDCs to gain access and accumulate within the CNS parenchyma rather than in perivascular locations.

### A High Level of Chimerism Alone Is Not Sufficient to Produce BMDC Accumulation within the Lumbar Spinal Cord of mSOD Mice

**Figure 4:**
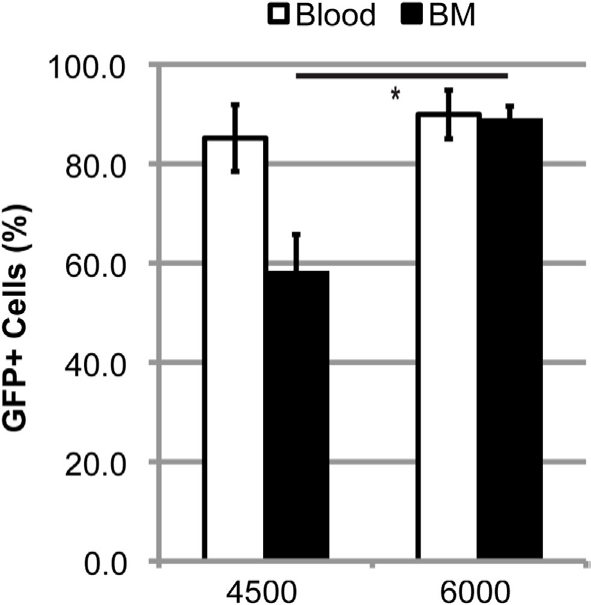
**High levels of blood and bone marrow (BM) chimerism are achieved in TREO-conditioned mice**. Flow cytometric quantification of GFP^+^ cells within the blood and BM in late stage mutant SOD1 mice that were conditioned with 4,500 or 6,000 mg/kg TREO and transplanted with GFP^+^ donor BM cells at 6–8 weeks of age. Data = mean ± SEM for *n* ≥ 3 mice/group; data were analyzed by a Student’s *t*-test with *p* < 0.05.

Doses of 60–125 mg/kg BU all led to similar levels of blood and BM chimerism, whereas the extent of BMDC accumulation within the lumbar spinal cord was different, suggesting that a high degree of chimerism alone is not sufficient to result in donor BMDC accumulation within the CNS, and that conditioning of the BBB/CNS is also necessary. To further explore this possibility, we established BM chimerism in mice by conditioning with TREO, a hydrophilic analog of BU that does not readily cross the BBB and enter the CNS. Conditioning with 6,000 mg/kg TREO generated a high level of chimerism comparable to that achieved with BU in both the blood and BM, whereas 4,500 mg/kg TREO resulted in a high level of chimerism in the blood but significantly lower levels of chimerism in the BM (Figure 4). Interestingly, TREO conditioning resulted in low levels of BMDC accumulation within the lumbar spinal cord of mSOD mice that were comparable to the levels of BMDC accumulation seen in the lumbar spinal cord of WT mice conditioned with BU (Table 1). Moreover, few stellate and ameboid BMDCs were detectable in the CNS of TREO-conditioned mice, suggesting that the majority of GFP^+^ cells were not localized within the CNS parenchyma.

### Mobilization of BM Cells into the Circulation Does Not Increase GFP^+^ BMDC Accumulation within the Lumbar Spinal Cord

**Figure 5:**
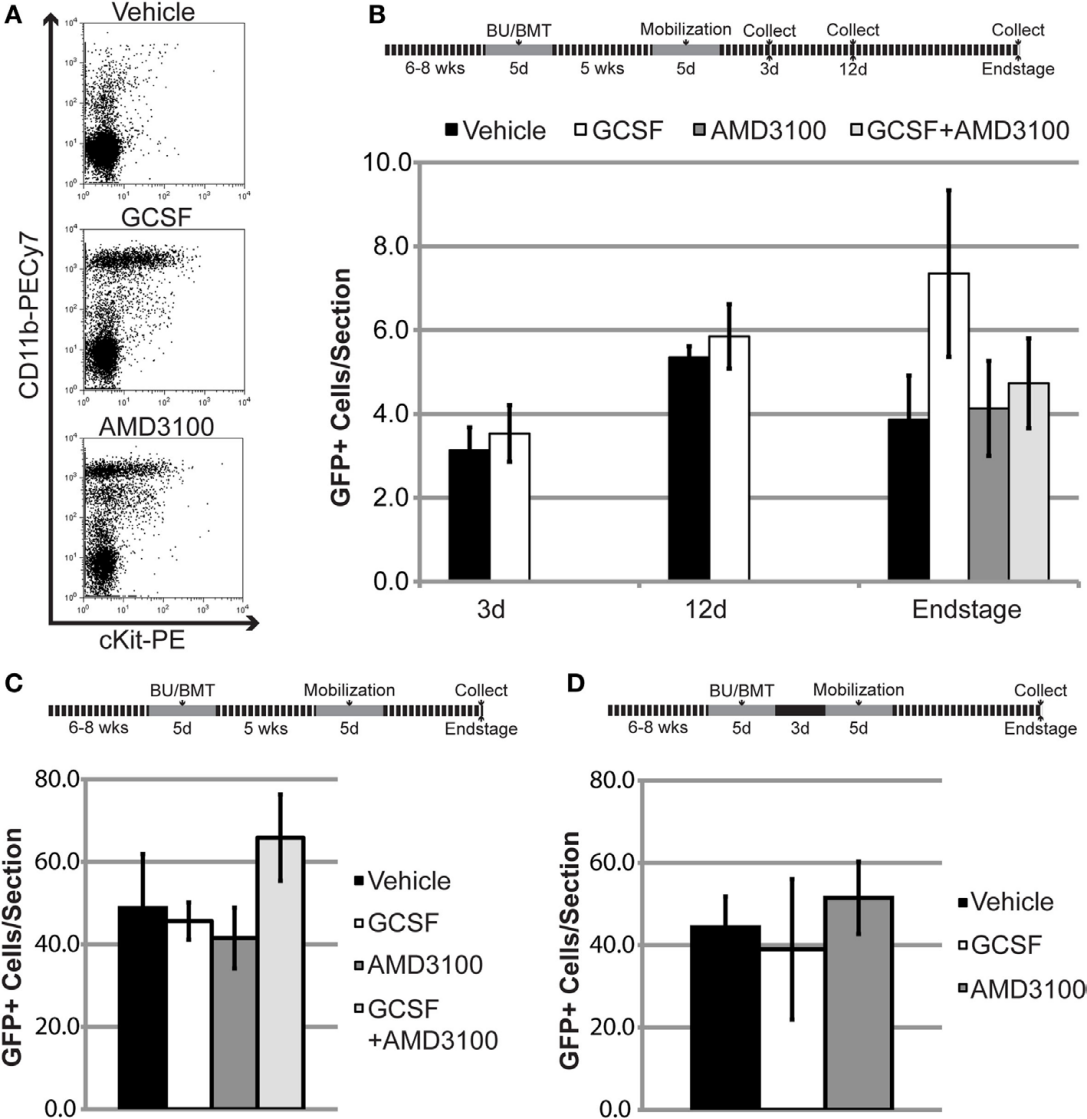
**Mobilization of bone marrow-derived cells (BMDCs) into the circulation does not increase BMDC accumulation within the lumbar spinal cord of mutant SOD1 (mSOD) mice and wild-type (WT) controls**. **(A)** Flow cytometric analysis of peripheral blood following mobilization treatments in WT mice. Results are representative images from *n* = 3 mice. **(B)** The 6- to 8-week-old WT mice were conditioned with 80 mg/kg of BU and transplanted with GFP^+^ donor bone marrow (BM) cells. Chimerism was allowed to establish for 5 weeks prior to mobilization treatment. GFP^+^ cells were quantified in lumbar spinal cord sections at the indicated timepoints. Data = mean ± SEM for *n* ≥ 3 mice/treatment. **(C,D)** The 6- to 8-week-old mSOD mice were conditioned with 80 mg/kg of BU and transplanted with GFP^+^ donor BM cells. Chimerism was allowed to establish for 5 weeks **(C)** or 3 days **(D)** prior to mobilization treatment. GFP^+^ cells were quantified in lumbar spinal cord sections at disease end stage. Data = mean ± SEM for *n* ≥ 4 mice/treatment.

Work by Ajami et al. using parabiotic mice suggests that the BM cell type(s) capable of entering the CNS following conditioning and BMT are primitive cells typically restricted to the BM (14). Under physiological conditions, primitive BM cells/progenitors are retained within the BM by stromal cell-derived factor-1 (SDF1), a chemokine with chemoattractant properties for hematopoietic cells expressing the complementary receptor CXCR4 (29). BMDCs were transiently mobilized from the BM into the circulation using either GCSF, a cytokine frequently used clinically to indirectly disrupt SDF1–CXCR4 binding, or plerixafor (AMD3100), a small molecule that directly inhibits the SDF1–CXCR4 interaction (Figure 5A) (29).

To determine if mobilized BMDCs could accumulate in the CNS and increase GFP^+^ cell number, high levels of stable BM chimerism (>85% GFP^+^ cells) were established for 5 weeks in mice conditioned with 80 mg/kg BU. However, mobilization with either GCSF or AMD3100 did not increase GFP^+^ cell number within the lumbar spinal cord in late-stage mSOD mice and age-matched WT controls (Figures 5B,C). We also co-administered GCSF and AMD3100, as gene expression analyses suggest that this combination mobilizes different cell types compared to single drug treatment (30). However, use of both drugs did not result in increased GFP^+^ number within the lumbar spinal cord in late-stage mSOD mice and age-matched WT controls (Figures 5B,C). As evidence suggests that BMDC accumulation within the CNS is dependent upon conditioning of the BBB/CNS, we also performed experiments where cells were mobilized 3 days post-BMT in mSOD mice (Figure 5D), a timepoint where we hypothesized that the effects of BBB/CNS conditioning would still be present. Even when BM cells were pharmacologically mobilized at this early timepoint, there was no increase in the number of BMDCs accumulating in the lumbar spinal cord of treated mice (Figure 5D).

### BMDCs Accumulate in the CNS More Rapidly in Mice Conditioned with Higher Doses of BU

**Figure 6:**
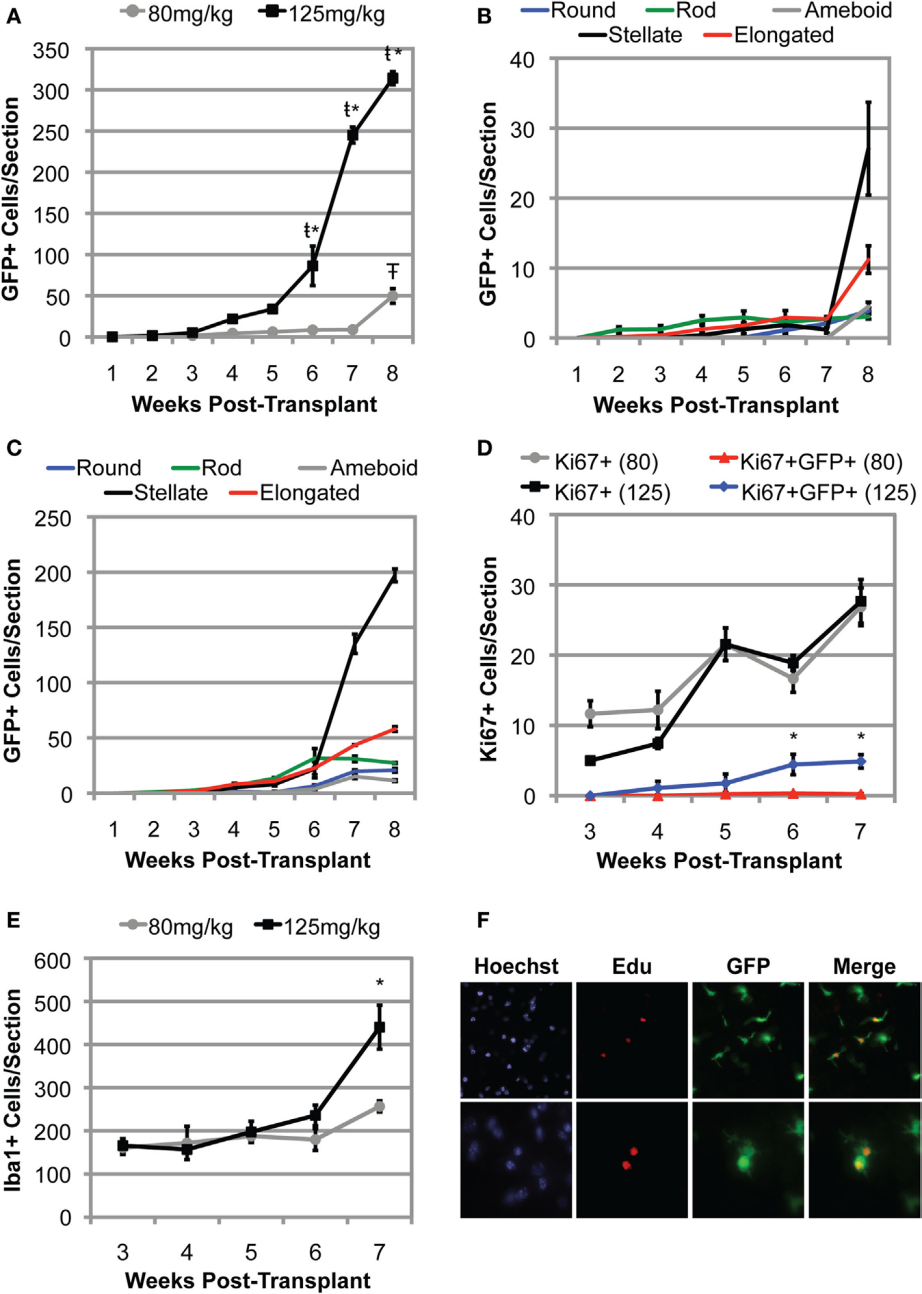
**Bone marrow-derived cells accumulate more rapidly and to a greater extent in mutant SOD1 (mSOD) mice conditioned with a high dose of BU compared to a lower dose**. **(A)** Weekly quantification of GFP^+^ cells within the lumbar spinal cord in mSOD mice conditioned with either 80 or 125 mg/kg BU and transplanted at 15 weeks of age. Data = mean ± SEM for *n* = 3 mice/timepoint. **(B)** Weekly quantification of GFP^+^ cell morphology within the lumbar spinal cord in mSOD mice conditioned with 80 mg/kg BU and transplanted at 15 weeks of age. Data = mean ± SEM for *n* = 3 mice/timepoint. **(C)** Weekly quantification of GFP^+^ cell morphology within the lumbar spinal cord in mSOD mice conditioned with 125 mg/kg BU and transplanted at 15 weeks of age. Data = mean ± SEM for *n* = 3 mice/timepoint. **(D)** Weekly quantification of Ki67^+^ cells and GFP^+^Ki67^+^ cells within the lumbar spinal cord in mSOD mice conditioned with either 80 or 125 mg/kg BU and transplanted at 15 weeks of age. Data = mean ± SEM for *n* = 3 mice/timepoint. **(E)** Weekly quantification of Iba1^+^ cells within the lumbar spinal cord in mSOD mice conditioned with either 80 or 125 mg/kg BU and transplanted at 15 weeks of age. Data = mean ± SEM for *n* = 3 mice/timepoint. **(F)** Immunohistochemical analysis of lumbar spinal cord sections from late stage mSOD mice that were conditioned with 125 mg/kg BU and transplanted with GFP^+^ donor BM cells at 15 weeks of age. Mice were given IP injections of 50 mg/kg 5-ethynyl-2′-deoxyuridine (EdU) 48 and 24 h prior to collection to label proliferating cells. Nuclei are shown in blue, EdU in red, and GFP^+^ cells in green. Results are representative images from *n* = 3 mice. Data were analyzed by ANOVA, and *post hoc* tests using the Tukey–Kramer method were used. * represents a significant difference between 80 and 125 mg/kg treatment groups with *p* < 0.01; ŧ represents a significant difference from the preceding timepoint with *p* < 0.01; Ŧ represents a significant difference from the preceding timepoint with *p* = 0.02.

To better understand the kinetics of BMDC accumulation within the CNS, we conditioned 15-week-old recipient mSOD mice with either low dose (80 mg/kg) or high dose (125 mg/kg) BU prior to transplantation with GFP^+^ BM cells. Transplanted mice were collected weekly, and the number of GFP^+^ BMDC cells accumulating within the lumbar spinal cord was quantified. During the first 3 weeks post-BMT, very few cells were detectable in the lumbar region of the spinal cord, regardless of the BU dose used for conditioning (Figure 6A). Moreover, the GFP^+^ cells that were detected were primarily rod shaped, with no stellate cells identified, suggesting that these GFP^+^ BMDCs had not yet accumulated within the CNS parenchyma (Figures 6B,C). Interestingly, the number of GFP^+^ BMDCs accumulating within the lumbar spinal cord of mSOD mice conditioned with 80 mg/kg of BU remained low for 7 weeks post-BMT and only showed a significant increase when the mice were approaching disease end stage 8 weeks post-BMT (Figure 6A). Conversely, the number of BMDCs accumulating within the lumbar region of the spinal cord in mice conditioned with 125 mg/kg BU showed significant increases at weeks 6–8 post-transplant (Figure 6A). Importantly, a large proportion of these GFP^+^ BMDCs accumulating within the lumbar spinal cord had a stellate morphology (>50% of BMDCs at weeks 7 and 8 post-BMT), suggesting that these cells were localized within the CNS parenchyma (Figure 6C).

### BMDC Accumulation within the Lumbar Spinal Cord Is Due in Part to Local Proliferation

To determine whether the accumulation of BMDCs in the lumbar spinal cord was due to proliferation of the GFP^+^ BMDC population, we immunolabeled dividing cells with Ki67, a nuclear protein expressed during mitosis (31). In spinal cords from mice treated with 80 mg/kg BU, Ki67^+^ cells were all GFP^−^, except for rare GFP^+^Ki67^+^ cells, indicating that proliferation of the BMDC population was very limited at all timepoints between weeks 3 and 7 post-transplant (Figure 6D). In spinal cords from the 125 mg/kg BU group, there were small numbers of GFP^+^Ki67^+^ cells at all timepoints from 4 to 7 weeks post-transplant (Figure 6D). The number of GFP^+^Ki67^+^ cells increased significantly in weeks 6 and 7 post-transplant, but cell numbers were small (approximately 5 cells/spinal section; Figure 6D). Interestingly, the increase in GFP^+^Ki67^+^ cells paralleled the increase in BMDC accumulation within the lumbar spinal cord (Figures 6A,D). Moreover, many of the GFP^+^Ki67^+^ and GFP^+^EdU^+^ BMDCs in the lumbar spinal cord exhibited a stellate morphology (Figure 6F), suggesting these cells were located in the CNS parenchyma. By contrast, GFP-Ki67 cells were much more evident at all timepoints. These data demonstrate some proliferation of GFP^+^ cells in spinal cord conditioned with 125 mg/kg BU, but that the extent of proliferation of these BMDCs may be limited.

We also evaluated the numbers of Iba1^+^ cells in spinal cord for both the 80 and 125 mg/kg BU groups and found that cell numbers did not increase significantly between weeks 3 and 6 (Figure 6E). At week 7, we found a significant increase in Iba1^+^ cells in the 125 mg/kg BU group, but not the 80 mg/kg mice (Figure 6E), suggesting that the more extensive conditioning of 125 mg/kg BU may increase the Iba1 cell number. The majority of Iba1^+^ cells in the lumbar spinal cord of mice conditioned with 125 mg/kg BU were GFP^−^, measuring 98.5, 91.5, and 72.2% at weeks 3, 5, and 7 post-transplant, respectively. While GFP^+^ cells comprised little of the Iba1^+^ population at weeks 3 and 5 post-transplant (1.5 and 8.5%), by 7 weeks post-transplant GFP^+^ cells were 27.8% of the Iba1^+^ population.

## Discussion

### Conditioning with BU or TREO Produces High Levels of Stable BM Chimerism

Establishment of BM chimerism in animals has typically been achieved using myeloablative doses of irradiation. However, irradiation can lead to secondary complications and alternative approaches to achieving adequate conditioning for BMT have thus been explored (1). We have previously shown that a high degree of BM chimerism can be achieved using myelosuppressive conditioning with the chemotherapeutic molecule BU (3, 11, 12), and these results are supported by several other laboratories (4, 8–10). Consistent with these findings, we have now shown that conditioning with a higher dose of BU (125 mg/kg) followed by BMT leads to rapid establishment of chimerism exceeding 90% in the BM of recipient mice. Moreover, the BM chimerism achieved using BU conditioning is stable, with high levels maintained for at least 1 year post-BMT. Similarly, conditioning with the BU analog TREO (6,000 mg/kg) produces a high degree of BM chimerism in mice comparable to BU (Figure 4). Our findings are similar to those of Van Pel et al. and Nasa et al., who have shown that concentrations of TREO ≤4,500 mg/kg result in lower, variable levels of chimerism, while 6,000 mg/kg TREO resulted in chimerism of ~80% (22, 23).

These levels of chimerism were achieved when using syngeneic recipients. However, when recipients are not syngeneic, modifications of this conditioning protocol may be needed. For example, in a recent study evaluating the accumulation of BMDCs in the CNS of a murine model of Alzheimer’s disease (AD), we transplanted C57BL/6 GFP^+^ BM into mice having a mixed 129/C57Bl/6 background. No sustained chimerism was achieved unless anti-asialo ganglio-*N*-tetraosylceramide antibody was used to deplete natural killer cell activity, likely due to “hybrid resistance” caused by autoreactive natural killer cells from the mixed background recipient being directed to the transplanted BM (12). With BMT into syngeneic mice, no graft rejection was observed.

### CNS Conditioning Is Required for BMDC Accumulation within the CNS

High levels of BM chimerism (>85%) were achieved using BU doses of 60–125 mg/kg. However, despite having comparable levels of chimerism, BMDCs accumulated within the lumbar spinal cord in a dose-dependent manner. These results indicate that establishment of high levels of chimerism alone does not result in the accumulation of BMDCs within the CNS. In addition, when high levels of BM chimerism were achieved using TREO, low numbers of BMDCs were detectable within the lumbar spinal cord. Based upon its hydrophilicity, TREO would not be predicted to readily cross the BBB and gain access to the CNS. In support of this claim, little penetrance of TREO and its active metabolites were detectable within the CNS of rats administered 500 mg/kg TREO, and limited amounts of TREO crossed an *in vitro* BBB model (20, 21). Virtually, all of the BMDCs detectable in TREO-conditioned mice had morphologies suggesting blood vessel-associated localizations (Table 1). The very small proportion of the BMDCs with stellate or ameboid morphologies detected within the lumbar spinal cord of TREO-conditioned mice (<2%) is likely due to limited TREO penetration of the BBB and CNS conditioning. These results suggest that some degree of BBB disruption and/or CNS conditioning also likely needs to be present, either by irradiation or chemotherapy. BU has been shown to cross the BBB (~20% of a dose administered in humans) (32), and evidence suggests that the BBB is not obviously disrupted by BU conditioning (10). In crossing the BBB, BU may be creating niche space within the microglial compartment allowing for BMDCs to infiltrate the CNS and accumulate (8). Alternatively, as high doses of BU have been shown to have neurotoxic effects in patients and animals (33), it is also possible that BU is causing neuronal damage, which in turn increases BMDC accumulation through increased recruitment of BMDCs from the circulation and/or stimulating local BMDC proliferation within the CNS. Unlike the findings of Butovsky et al. (18), the present data indicate that in the absence of a CNS conditioning stimulus, there is limited entry and accumulation of BMDCs in the spinal cord, even in diseases such as murine ALS.

### BMDC Accumulation within the CNS Is Dependent on Disease-Related Mechanisms

Small numbers of BMDCs were observed in WT mice conditioned with BU. Moreover, the morphology of BMDCs accumulating within the lumbar spinal cord of WT mice suggest that few, if any, of the cells were located within the CNS parenchyma. These GFP^+^ BMDCs were likely perivascular, even when 125 mg/kg BU was used. Recent studies by Wilkinson et al. found that 125 mg/kg BU caused considerable accumulation of stellate BMDCs within the CNS of WT mice, an effect that was larger than that caused by myeloablative irradiation (4). Using the same dose of BU conditioning, we found substantially lower numbers of GFP^+^ BMDCs in the lumbar spinal cord and fewer GFP^+^ cells with a stellate morphology than Wilkinson and colleagues (Table 1). The differences we see in BMDC accumulation compared to Wilkinson et al. may be due to different CNS tissue being analyzed (brain vs spinal cord) or a slightly different transplantation procedures where we transplanted 10-fold less donor BMDCs (4).

As in many previous studies using both irradiative and non-irradiative conditioning, we find much larger numbers of BMDCs in the lumbar spinal cord of mSOD mice compared to WT mice, an effect that is also seen in other models of neurodegenerative disease (Table 1; Figure 3) (3, 11, 12). Stellate and ameboid cells generally constitute parenchymal cells within the CNS (28) and cells with these morphologies were frequently seen in mSOD mice, while very few were observed in WT mice, suggesting that disease-related mechanisms were involved in BMDC accumulation within the CNS. Notably, the majority of BMDCs in the lumbar spinal cord of late stage mSOD mice conditioned with 125 mg/kg BU had a stellate morphology (Table 1), while our previous work using myeloablative irradiation showed the majority of cells to have an ameboid morphology (3). These differences in BMDC morphology between irradiation and BU may be related to the cytokine profile triggered by the conditioning treatment (3).

From previous work, it is known that the BMDCs that accumulate in the CNS are mostly monocytic lineage cells that express CX_3_CR1, although small numbers of T lymphocytes may be present (2, 27, 34, 35). It is likely that endogenous microglia and monocyte-derived CNS macrophages constitute distinct populations with different functions (36, 37), but that the properties of these cell populations will differ in neurodegenerative disorders such as ALS or diseases with prominent inflammation, such as experimental autoimmune encephalitis (EAE). In EAE, monocyte lineage cells efficiently enter the CNS and give rise to mature macrophages that seem indistinguishable morphologically from resident microglia (38). However, as recovery from the acute phase of EAE occurs, infiltrating monocyte lineage cells which had accumulated in the CNS undergo apoptosis (38). In contrast, the extent of BMDC accumulation in models of chronic neurodegenerative diseases such as ALS increases, likely due to expansion of accumulated BMDCs and possibly further BMDC entry. The factors responsible for BMDC entry include CCR2, as limited BMDC accumulation of BMDC occurs in CCR2^−/−^ mice (38). Based upon work in EAE, it has been claimed that monocyte-derived macrophages are phagocytic and inflammatory, whereas resident microglia have less inflammatory properties based on gene expression data (37). For instance, monocytes demonstrate an upregulation of CXCR2, CCR1, toll-like receptor 6, and other genes associated with inflammation, whereas microglia upregulate TNF, Stat1, and other genes associated with metabolism (37). It is likely that the method of chimerism used to achieve BMT in such studies will be relevant for the profile of gene expression observed. In mice transplanted using BU, elevations are seen in levels of serum and CNS G-CSF and IL-6 shortly following BMT, compared to irradiation (4). Monocyte lineage cells ultimately derive from the monocyte/macrophage and dendritic cell precursor (MDP) (13). There is also evidence for a clonogenic monocyte/macrophage restricted progenitor cell derived from the MDP termed the common monocyte progenitor (39) which could, in principle, be a cell type that accumulates in the CNS following BU administration and BMT.

### Mobilization of BM Cells into the Circulation Does Not Increase BMDC Accumulation in the CNS

During BMT using whole or fractionated BM, BM cells including progenitors are injected into the circulation of the recipient animal and may accumulate within the CNS. In an attempt to increase BMDC accumulation within the CNS, we mobilized cells from the BM using GCSF, AMD3100, or a combination of both treatments. Despite mobilizing BM cells (Figure 5A), we were unable to increase the amount of BMDC accumulation in the lumbar spinal cord. It is possible that the BM cells mobilized by GCSF and AMD3100 were not capable of migrating into the CNS. Alternatively, BM cells in the blood circulation may not have been mobilized for sufficient time, or in sufficient numbers to accumulate within the CNS. While there is evidence showing that GCSF can increase the number of BMDCs accumulating in the CNS of a mouse model of AD, these treatments were typically combined with other factors such as SDF-1, which would be expected to influence BMDC migration (26, 40). Furthermore, these experiments were performed in irradiated mice which may lead to different CNS/BBB conditioning that allows for the accumulation of mobilized cells within the CNS. These observations have implications for the treatment of ALS. Previous studies have used GCSF treatment in ALS patients to produce BM mobilization for collection and re-injected into patients to in an attempt to generate BMDC accumulation in the CNS (41, 42). However, given the present results, it remains doubtful as to whether GCSF treatment will alter BMDC accumulation in the CNS.

### BMDC Accumulation and Proliferation

The weekly analysis of BMDC accumulation within the lumbar spinal cord indicates that following myeloablation and chimerism, only limited cell accumulation occurs for several weeks. A substantial increase in cell accumulation occurs only after the first 5 weeks post-transplant in mSOD mice conditioned with 125 mg/kg BU and after 7 weeks in mice conditioned with 80 mg/kg BU. This increase in cell number corresponds to a time when mice are developing neurological deficit, raising the possibility that the increased cell number occurs in relation to disease-related signals. It is well known that microglial proliferation occurs in mSOD mice (43). It is possible that the GFP^+^ BMDCs that seeded the CNS proliferate at a similar rate of expansion as endogenous microglia and that this expansion is responsible for some of the large numbers of GFP^+^ BMDCs seen, especially in mSOD mice at later times following transplantation (23). However, the present results show only small numbers of Ki67^+^GFP^+^ cells suggesting that in addition to cell proliferation, some entry of BMDCs likely occurs and that this entry is likely dependent on the conditioning stimulus.

It remains unclear to what extent BMDC accumulation within the CNS of BU-conditioned mice is due to proliferation compared to continued infiltration. In experiments using the proliferation markers EdU or Ki67, we found cell labeling of stellate GFP^+^ cells within the lumbar spinal cord. However, the number of cells labeled with these markers was small, consistent with the findings of Wilkinson et al. (4). As we only administered EdU for 48 h prior to collecting tissue, it is unlikely that the cells divided in the BM and subsequently migrated into the lumbar spinal cord parenchyma. Moreover, several of the EdU-labeled cells we observed appeared to be actively dividing (Figure 6F), and Ki67 only labels cells that are proliferating. The limited numbers of cells labeled by EdU is likely a consequence of the short-time course that EdU can be administered to minimize the possibility of labeling proliferating within the BM that could subsequently infiltrate the lumbar spinal cord. Moreover, Ki67 only labels cells that are actively proliferating at the time of fixation and tissue collection. Thus, it is likely these techniques underestimate the extent that proliferation accounts for BMDC accumulation within the CNS.

## Conclusion

The use of BMDCs in the therapy of CNS disease is in its infancy. To be clinically useful, BMDCs will need to be administered with conditioning agents that are mild enough to be tolerated, yet permit long-term BM chimerism. BU appears to be useful for this aim. Furthermore, this work and others have shown that high-level blood and BM chimerism are insufficient to ensure that large numbers of BMDCs will accumulate in the CNS. The results also show that substantially more BMDC accumulation can occur in a disease model than in WT animals, indicating that disease-related factors will influence BMDC entry, likely in a time-dependent manner. We do not find that mobilization of BM by GCSF or AMD3100 influences the extent of BMDC accumulation in CNS but suggests that BMDCs that accumulate in the CNS increase in number partially through proliferation.

## Author Contributions

KP, JM, C-AL, FR, and CK conceptualized the experiments. All experiments were performed by KP with the assistance of JM and KT. The manuscript was written by KP and revised by C-AL, FR, and CK.

## Conflict of Interest Statement

The authors declare that the research was conducted in the absence of any commercial or financial relationships that could be construed as a potential conflict of interest.
